# Evidence for morph-specific substrate choice in a green-brown polymorphic grasshopper

**DOI:** 10.1093/beheco/arab133

**Published:** 2021-12-16

**Authors:** Pauline Heinze, Petra Dieker, Hannah M Rowland, Holger Schielzeth

**Affiliations:** 1 Population Ecology Group, Institute of Ecology and Evolution, Friedrich Schiller University Jena, Dornburger Straße, Jena, Germany; 2 Research Group Predators and Toxic Prey, Max Planck Institute for Chemical Ecology, Hans-Knöll-Straße, Jena, Germany

**Keywords:** Acrididae, background choice, balancing selection, color polymorphism, Gomphocerinae, matching habitat choice, microhabitat choice, Orthoptera, visual modeling

## Abstract

Orthopteran insects are characterized by high variability in body coloration, in particular featuring a widespread green-brown color polymorphism. The mechanisms that contribute to the maintenance of this apparently balanced polymorphism are not yet understood. To investigate whether morph-dependent microhabitat choice might contribute to the continued coexistence of multiple morphs, we studied substrate choice in the meadow grasshopper *Pseudochorthippus parallelus.* The meadow grasshopper occurs in multiple discrete, genetically determined color morphs that range from uniform brown to uniform green. We tested whether three common morphs preferentially choose differently colored backgrounds in an experimental arena. We found that a preference for green backgrounds was most pronounced in uniform green morphs. If differential choices improve morph-specific performance in natural habitats via crypsis and/or thermoregulatory benefits, they could help to equalize fitness differences among color morphs and potentially produce frequency-dependent microhabitat competition, though difference appear too small to serve as the only explanation. We also measured the reflectance of the grasshoppers and backgrounds and used visual modeling to quantify the detectability of the different morphs to a range of potential predators. Multiple potential predators, including birds and spiders, are predicted to distinguish between morphs chromatically, while other species, possibly including grasshoppers themselves, will perceive only differences in brightness. Our study provides the first evidence that morph-specific microhabitat choice might be relevant to the maintenance of the green-brown polymorphisms in grasshoppers and shows that visual distinctness of color morphs varies between perceivers.

## Teaser

Grasshoppers are highly variable in body color. But do individuals of different color variants choose different substrates to rest on? We show that they do. Furthermore, we test if potential predators are able to perceive the differences between color variants. It turns out that some do and some do not. Individual substrate choice can thus contribute to the coexistence of different color variants within the same species in the same area.

## INTRODUCTION

A fundamental question in evolutionary biology is which processes drive the origin and maintenance of polymorphisms (Jamie and Meier 2020; Orteu and Jiggins 2020). Color polymorphism is defined as the sympatric coexistence of multiple discrete color variants in interbreeding populations independent of sex, age, and other state-dependent modifiers (Ford 1945). Multiple mechanisms can contribute to the balanced maintenance of color polymorphisms, including spatially heterogeneous selection in populations connected by gene flow (Hedrick et al. 1976; Hedrick 2006; Gordon et al. 2015), temporally fluctuating selection (Siepielski et al. 2009; Bell 2010), pleiotropic fitness trade-offs across different contexts (Roff and Fairbairn 2007), disassortative mating preferences (Roulin and Bize 2007; Wellenreuther et al. 2014), and negative-frequency dependent selection (for example by predators that form search images, Bond and Kamil 1998, 2006; Bond 2007; Ruxton et al. 2019). But also matching habitat choice can contribute to the maintenance of phenotypic polymorphisms by equalizing fitness differences. The relevance of different mechanisms can vary between study systems, which calls for a careful analysis of each individual case.

Color polymorphisms are widespread across the animal kingdom, but they are usually limited to few species within clades (Hugall and Stuart-Fox 2012). The green-brown polymorphisms in polyneopteran insects represent one of the lesser known, yet very penetrant color polymorphisms (Rowell 1972; Dearn 1990). The insect order Orthoptera, which includes grasshoppers, crickets, and bush crickets, is particularly remarkable in that the green-brown polymorphism is present in a large proportion of species (e.g. 45% of East African acridid grasshoppers, Rowell 1972, 30% of all European Orthoptera, Schielzeth 2020). The two orthopteran suborders (Caelifera, Ensifera) separated about 330 million years ago (Song et al. 2020) and both suborders contain many color-polymorphic species. The widespread occurrence of the green-brown polymorphism in Orthoptera is indicative of balancing selection contributing to its maintenance. Balancing selection describes any selective process that maintains polymorphism and includes in particular negative frequency-dependent selection (Hedrick 2007). Some other groups in the Polyneoptera (e.g. the Mantodea, Phasmatodea, and Mantophasmatodea, which have diverged from Orthoptera about 380 Mya, Song et al. 2020) feature a phenotypically similar green-brown polymorphism (e.g. Roth et al. 2014; Comeault et al. 2016). This calls for explanations for how this specific color polymorphism is maintained in this clade of insects.

Orthopterans are known for their frequent homochromy that is the observation that local populations are dominated by individuals matched to local backgrounds (Rowell 1972). Homochromy, in a classical sense, is a feature at the level of populations, but individual-level decision such as background choice behaviors might well contribute to such sorting processes. Local patches often show spatial heterogeneity, due to the uneven distribution of moisture and nutrients, producing microhabitat differences within the home range of individuals. There are multiple processes that can lead to homochromy, in particular, local adaptation by natural selection across generations, selective mortality within generations (Forsman and Appelqvist 1998; Civantos et al. 2004), background-dependent phenotypic plasticity (Rowell 1972; Edelaar et al. 2017; Peralta-Rincon et al. 2017), and matching habitat choice (Edelaar et al. 2008; Edelaar et al. 2019). Among orthopterans, homochromy has mostly been studied in the ground-dwelling species of the subfamily Oedipodinae, in which coloration varies in darkness and tone with local soil types (Rowell 1972; Baños-Villalba et al. 2018), and in the Tetrigidae that vary in darkness and pattern (Hochkirch et al. 2008). Most reports on homochromy in Orthoptera refer to variability in color tone (the orange-black pigmentation system dominated by ommochromes and melanins, Rowell 1972), while much less is known about homochromy in the green-brown pigmentation system (putatively controlled by biliverdin, Rowell 1972). It is unknown, in particular, if green-brown polymorphic species have a behavioral preference for different microhabitats.

Matching habitat choice has the potential to increase morph-dependent crypsis by decreasing the contrast between an animal’s body color and the substrate. Matching habitat choice has been studied in several orthopterans that are variable in overall body coloration, but none of these cases refers to the green-brown polymorphism. For example, the oedipodine grasshopper *Sphingonotus azurescens* has been found to show phenotypic plasticity in body color and matching habitat choice (Edelaar et al. 2017; Baños-Villalba et al. 2018). Phenotypic plasticity can co-occur with genetic predisposition as in the case of *S. azurescens* (Edelaar et al. 2019). Furthermore, two groundhoppers, *Tetrix undulata* and *Tetrix subulata*, with genetically determined color morphs that differ in overall darkness show temperature-dependent habitat preferences (Forsman 2000; Ahnesjö and Forsman 2006; Karpestam et al. 2012). The observation of homochromy itself might also be indicative of matching habitat choice, although this is open to alternative explanations as introduced above.

**Figure 1:**
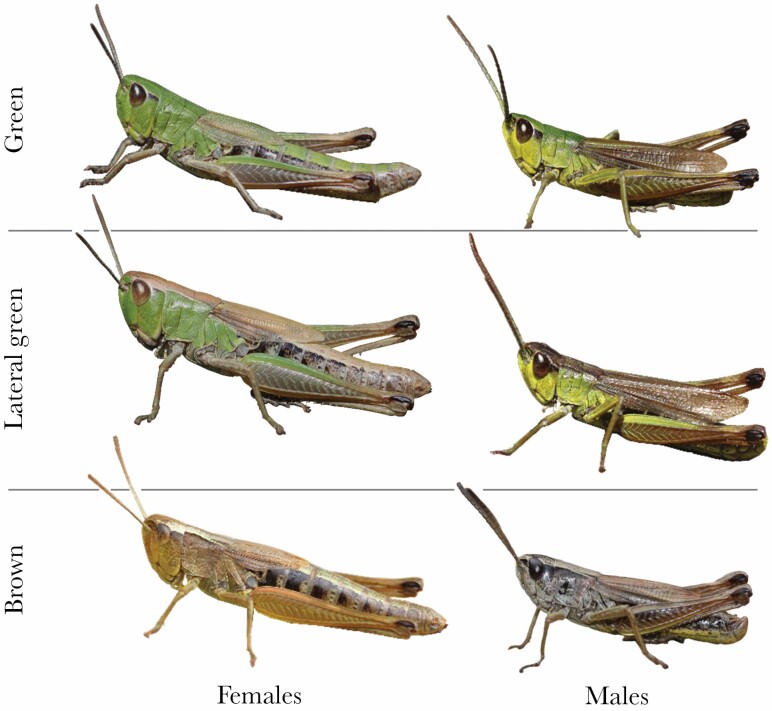
The three main color morphs of the adult meadow grasshopper *Pseudochorthippus parallelus* as used in this study. Images were taken under natural light conditions and serve for illustration of the general patterns. Late instar nymphae show the same color morphs and color morphs are stable throughout development once expressed from nymphal stage 2 or 3. Both sexes are usually flightless, because hind wings are vestigial.

Here we study substrate choice in wild-caught individuals of a gomphocerine grasshopper, the meadow grasshopper *Pseudochorthippus parallelus* (Zetterstedt 1821). This species lives in highly structured grasslands and perches on the vegetation as well as on the ground. Since the species is usually short-winged (mircopterous) and thus unable to fly (only a few percent of a population may develop long wings, Ingrisch and Köhler 1998), we limited our interest in behavioral choice to small spatial scales. There are five discrete color morphs in this species (Köhler et al. 2017), one of which is rare and rather subtle (brownish instead of green legs) while another one is markedly different in the distribution of green areas, but also rare in natural populations (Köhler et al. 2017). Most populations, therefore, consist of a mix of three common color morphs (uniform green, lateral green, and uniform brown; Figure 1). There is evidence that color morphs are genetically determined in this species and other species of the *Chorthippus* clade (Sansome and La Cour 1935; Köhler 2006; Winter et al. 2021), although it is possible that some individuals represent phenocopies, i.e. developmental variants that resemble genetic variants (West-Eberhard 2003).

We tested for differential preferences of different color morphs to rest on different substrate colors in the laboratory. We maintained individual grasshoppers on a checkered background (consisting of green and brown squares) from late nymphal stages into adulthood. The setup was intended to represent small-scale heterogeneous environments that characterize many grasslands. We used the three predominant morphs (uniform green, lateral green, and uniform brown) and predicted that with matching habitat choice, uniform green morphs would spend more time on green backgrounds, and uniform brown morphs more time on brown backgrounds. Bicolored, lateral green individuals that show a combination of green and brown areas were expected to show intermediate background preferences. Furthermore, we used spectrophotometry to measure the visual properties of the grasshoppers, and combined this with visual modeling to evaluate if potential predators and the grasshoppers themselves are able to perceive the color morph differences and background colors.

## MATERIALS AND METHODS

### Study species

We caught 125 meadow grasshoppers, *P. parallelus* (76 males, 49 females) from around Jena, Germany (50.95°N, 11.62°E) in May and June 2018. Grasshoppers were caught with sweep nets and all individuals in their second to fourth nymphal stage were collected and transferred to the laboratory. There are four nymphal stages plus the imago stage in this species and color morphs are distinct in later nymphal stages. Individuals are not known to switch between color morphs during ontogeny and no such case appeared in our study. Meadow grasshoppers are easily separated into uniform green individuals, lateral green individuals (upper side brown, sides green), and brown individuals (with complete absence of green; Figure 1; Köhler et al. 2017). A subtle morph that is green with brown legs was not used here, since the frequency of this morph is generally low in natural populations. This is also true for a morph with green dorsal stripe and brown sides (“dorsal green”) that was not included in our sample. In total, we collected a sample of 40 green individuals (16 females, 24 males), 51 lateral green individuals (19 females, 32 males), and 34 brown individuals (14 females, 20 males).

### Experimental setup

**Figure 2:**
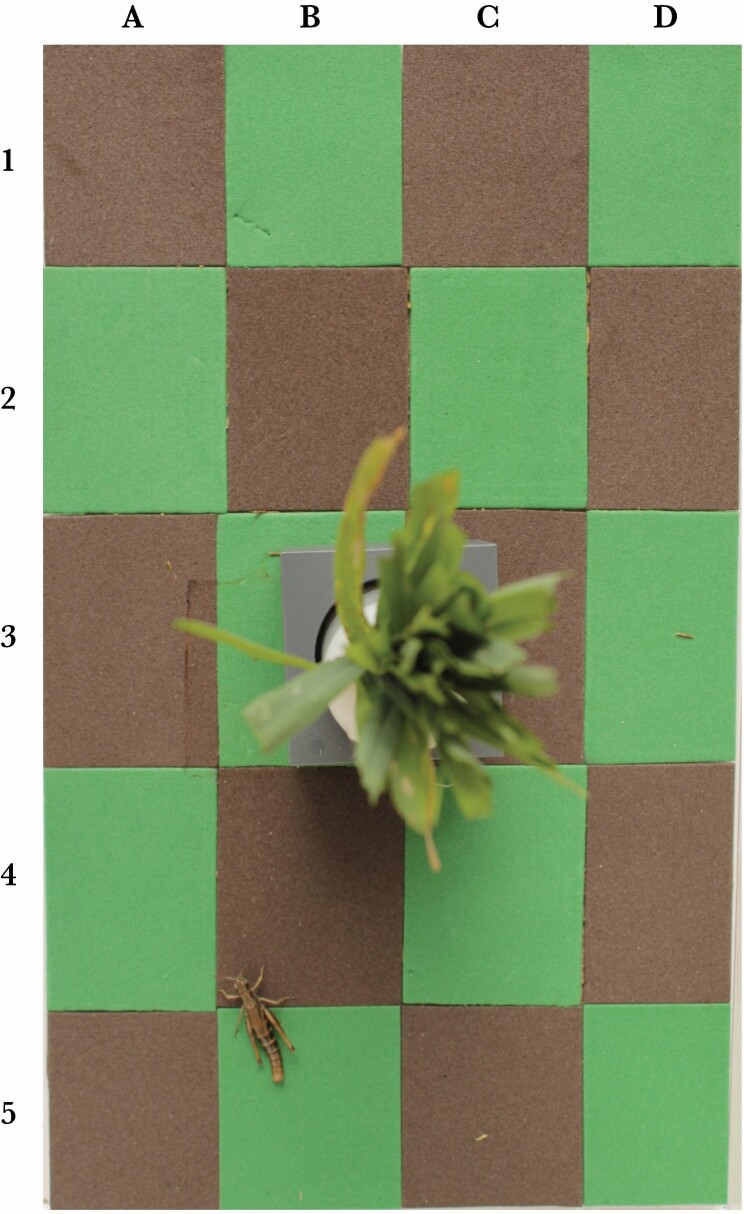
Experimental setup viewed from above. The size of the arena was 25 cm x 15 cm. The grass pot in the center was provided as food source. A brown morph female meadow grasshopper *Pseudochorthippus parallelus* is visible in patches B4/B5. The location of the head was recorded and analyzed (here B4).

Individuals were transferred to the laboratory where they were held in individual plastic cages of 25 cm x 15 cm x 15 cm (length x width x height) in size with air-permeable lids (Fauna Box Medium). The floor of each cage was lined with rubber foam in a 4 x 5 green-and-brown checkerboard patterns with 10 patches per color (Figure 2). We chose green and brown patches, because grasshoppers differ in green vs. brown morphs and because grasslands are heterogenous in green locations (fresh grass) and brown locations (bare ground or dry plant material). Experimental patches differed in color and brightness (see below), which is representative of natural habitats where patches of bare soil differ from vegetation not only in chroma, but also in brightness. Each patch was 3.75 cm x 5 cm in size. A white pot with cut grass (scintillation vials, 2.8 cm diameter, 6.1 cm height) in a gray socket of dimensions 4 cm x 4 cm was provided for food. Grass pots were placed in the middle of each cage in order to cover approximately equal amounts of the green and the brown central patches. Individuals were maintained from the day of capture until they died or till the experiment was terminated on 13^th^ August (25 ± 8 days, mean ± SD). Cages were placed in racks with illumination from above by full-spectral light tubes (WT120C G2 LED34S/840 PSD L1500, Philips). The surface temperature of green and brown patches was measured on six occasions with 2–4 pairs of patches measured per occasion (7 times in morning, 11 in afternoon) yielding a total of 18 pairs of measurements on five different days over a period of two weeks. We found no significant difference in temperatures (green: 29.72 ± 2.13°C, brown 29.72 ± 2.08°C, paired t test: *t*_17_ = –0.08, *P* = 0.93).

### Location data collection

Each individual’s position was recorded every 1–2 days (with one larger gap of 12 days) with 1–4 records per day. There were at least 1.5 hours between recordings (average interval between consecutive recordings on the same day 2.8 ± 1.4 hours). The position was recorded as the grid number and grid color. If the body of a grasshopper touched multiple patches, we used the location of the head as being the definitive patch choice. Occasions on which grasshoppers did not sit on the floor (e.g. they were often sitting on the grass, the walls, or under the lids) were recorded as missing data. Data collection ended when individuals died or until the experiment was terminated on 13^th^ August. The total recording period ranged from 29/05/2018 to 13/08/2018.

### Spectrophotometer measurements

Reflectance measurements of 60 adult individuals were taken with a hand-held spectrophotometer (Avantes, AvaSpec-2048, Eerbek, The Netherlands, Fiber FCR-7UVIR200-2-1.5X100 with 1.5 mm diameter) with a deuterium-halogen light source (Avantes, Ava-Light-D(H)-S). The device was calibrated with a commercial white standard (Avantes WS-2) each time before a new individual was measured. Measurements were taken perpendicular to the surface with the probe placed directly at the surface. The AvaSoft 7.5 software (Avantes, Eerbek, The Netherlands) was used for capturing spectra with integration time set to 100 ms and automatic averaging of five readings for one measurement. Ten males and ten females of each color morph were measured on the lateral lobes and on the dorsal side of the pronotum to account for predators that approach from the side and from above. We took five independent measurements on each of the two body areas and averaged reflectance across these five measurements yielding a total of 120 reflectance spectra (3 morphs x 2 sexes x 10 individuals x 2 areas). Furthermore, we measured ten green and ten brown experimental substrate patches five times each and averaged spectra per patch. Finally, we measured ten blades of fresh grass leaves and dry grass from the local habitat five times each on a black background. A narrow peak in reflectance between 554 and 660 nm appeared to represented an artifact of the device’s grating and was removed by replacement with the average across the range of 550–554 nm and 660–664 nm.

### Visual modeling

We used the R package *pavo* 2.4.0 (Maia et al. 2019) for the analysis of reflectance data. Reflectance data were analyzed for wavelengths in the range of 300–700 nm. We used the *addmin* option that adjusts for negative values by adding an offset to yield only non-negative values. No further optimizations were made. Spectra were aggregated by individual and body area. Color is not a property of any object but is a product of the visual and nervous system of the animal viewing the object (Endler 1990; Guilford & Dawkins 1991). We, therefore, used visual modeling for the objective assessment of how the focal animals’ appearance is perceived by the visual system of putative receivers.

We calculated color space coordinates and modeled the chromatic and achromatic contrasts between the three morphs for the visual systems of six species that are representative of potential predators: a trichromatic lizard (*Ctenophorus ornatus;* long wavelength-sensitive λ _max_ at 571 nm; medium wavelength-sensitive, λ _max_ 493 nm and short wavelength-sensitive, λ _max_ 440 nm SWS, Barbour et al. 2002), a trichromatic jumping spider (*Habronattus pyrrithrix*; UV sensitive, λ _max_ 377 nm; medium wave-sensitive, λ _max_ 530 nm; long wave-sensitive λ _max_ 626 nm, Zurek et al. 2015), a trichromatic hymenopteran insect (honey bee, *Apis mellifera;* short-wavelength sensitive, λ _max_ = 344 nm; medium-wavelength sensitive, λ _max_ = 436 nm; and long-wavelength sensitive; λ _max_ = 544 nm, Menzel and Backhaus 1991), a tetrachromatic dipteran insect (house fly, *Musca domestica*; *λ*_*max*_*at* 360, 420, 490, and 520 nm, Hardie and Kirschfeld 1983) and two tetrachromatic birds (European starling, *Sturnus vulgaris:* UV sensitive λ _max_ 362 nm; short-wavelength sensitive λ _max_ 449 nm; medium-wavelength sensitive λ _max_ 504 nm and long-wavelength sensitive λ _max_ 563 nm, Hart et al. 1998; and peafowl, *Pavo cristatus λ*_*max*_ at 432, 477, 537 and 605 nm, Hart 2002). Lizards, spiders, and birds are predators of grasshoppers (Ingrisch and Köhler 1998), and the two insects were chosen to represent predatory and parasitoid wasps and flies. Peak cone catch sensitivities of the honey bee are similar to peak sensitivities in the trichromatic migratory locust *Locusta migratoria* (Briscoe and Chittka 2001). However, since only peak sensitivities and not full sensitivity curves are available for *Locusta* we use the honey bee as the best proxy for grasshopper vision.

Visual models were implemented using flat, full-spectral illumination (“ideal” option in *pavo*) and a wavelength-independent background effect on color perception (“ideal” option in *pavo*), though the alternative choices of daylight illumination and vegetation backgrounds (as implemented in *pavo*) did not qualitatively affect the results. We calculated noise-weighted chromatic and achromatic visual distances among morphs using the receptor-noise model of Vorobyev et al. (1998) based on relative photoreceptor densities of the six animals that serves as representative for potential predicators (as implemented in the *coldist* function of *pavo*). For achromatic distances we used the starling double-cone option for starling, the chicken double-cone option for peafowl, the house fly R1-6 photoreceptor for the house fly, and the summed response of all photoreceptors for all other species. We used homogenous transmission (“ideal” option in pavo) and noise proportional to the Weber fraction (“neutral” option in pavo) when modeling visual distance. Similarly, we calculated visual distances between the sexes (separately by color morph and body parts), between the body parts, and between natural and artificial substrates. Finally, to evaluate crypsis, we calculated visual distances between grasshopper color and natural substrates.

### Statistical analysis

Substrate choice data were analyzed using generalized linear mixed model (GLMM) with binomial error distribution and logit link. We modeled the probability of sitting on green (rather than brown) patches as a binary response. All models fitted individual identity and date of recording as random effects to control for the nonindependence of data points. Missing data were excluded from the analysis. We first fitted a GLMM with only an intercept in the fixed part to estimate overall preferences for green vs brown backgrounds across morphs, sexes, and ages. Our main models aimed to test for morph differences in substrate choice and therefore fitted color morph (uniform brown, lateral green, uniform green) as fixed factors while controlling for sex and age (nymph vs. adult). Sex was coded as –0.5 and 0.5 for females and males, respectively, such that the estimate refers to the difference of males relative to females and the intercept estimates the effect averaged across the two sexes. Nymphal stage was coded 0 for adults and 1 for nymphae, such that the slope estimates the differences of nymphae relative to adults and the intercept refers to adults. Morph was coded as treatment contrasts with brown representing the reference category. The intercept thus refers to a brown adult averaged across sexes. We fitted the fixed-effect interactions age x morph and sex x morph and due to the way of coding, main effects are meaningfully interpretable even in the presence of interactions. The significance of random effects was tested by likelihood ratio tests (LRT) and the significance of fixed effects was tested by Wald tests. For plotting, we removed the intercept to get three estimates of mean preferences for the three color morphs (Schielzeth 2010). Mixed models were fitted in R 4.0.2 (R Core Team 2020) using the *glmer* function from the *lme4* package (version 1.1-23, Bates et al. 2015) and the rpt package (version 0.9.22, Stoffel et al. 2017) for estimating repeatabilities.

We also assessed whether random-slope models were required. Random slopes refer to the nonindependence of slopes at the level of any of the random effects and failure to account for variability in slopes might lead to false positives when estimating the population slope (Schielzeth and Forstmeier 2009). However, the main factors of interest (morph identity) is a group-level predictor with respect to individual and thus not subject to random-slope variation. We fitted a morph-by-date random-slope interaction, but this was not statistically significant (LRT: χ ^2^_5_ = 3.30, *P* = 0.65) as was the sex-by-date random-slope interaction (LRT: χ ^2^_2_ = 2.00, *P* = 0.37). Furthermore, random-slope interactions of age with either individual (LRT: χ ^2^_2_ = 2.33, *P* = 0.31) or date (LRT: χ ^2^_2_ = 0.38, *P* = 0.83) were both not statistically significant. None of these random-slope terms affected the conclusions or was of primary relevance to the central hypothesis. We, therefore, present results without random slopes.

## RESULTS

### Substrate choices

In total, we recorded 3650 positions of individual grasshoppers (2% in nymphal stage 3, 9% in nymphal stage 4, and 90% as adults). In 1348 instances (37%) grasshoppers were sitting on the floor so that patch color could be recorded, while in other cases grasshoppers were sitting on the grass, on the cage walls, or under the lids or could otherwise not be assigned to any patch color. We recorded 10.8 ± 8.3 (mean ± SD) valid observations per individual. There was an overall significant preference for green patches with 56% of all valid observations on green backgrounds (GLMM without fixed effects: β _0_ = 0.27 ± 0.07, *z* = 3.848, *P* = 0.00011).

**Figure 3:**
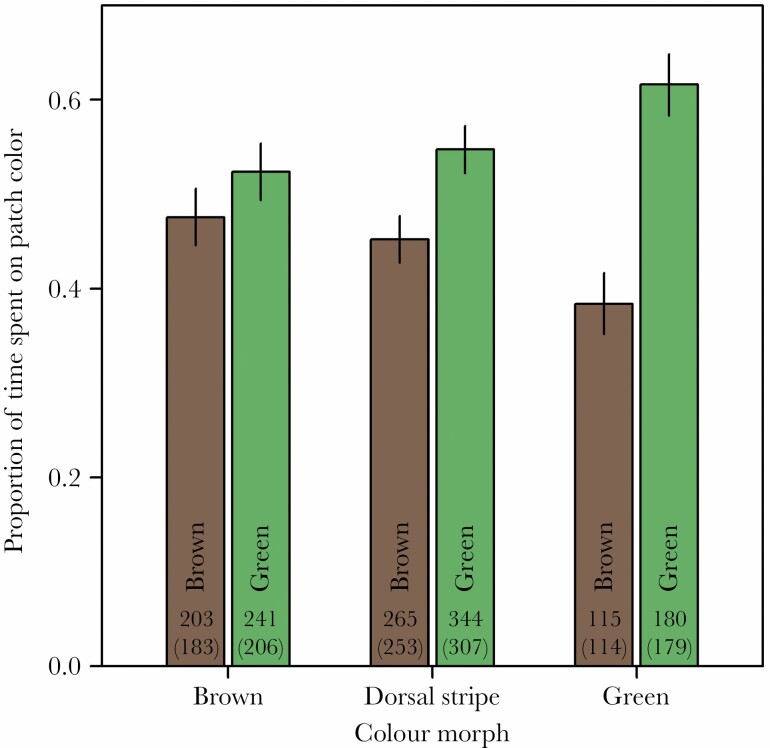
Background color preferences of three color morphs of the meadow grasshopper *Pseudochorthippus parallelus*. Estimates are from a mixed model with bars indicating SE and numbers show the number of records (the subset of records for imagoes is shown in brackets).

There were significant morph-specific differences in substrate choice (Figure 3). Uniform brown morphs did not significantly prefer any background color (54% of positions observed on green patches, β _0_ = 0.10 ± 0.12, *z* = 0.80, *P* = 0.42). Lateral green morphs tended to spend more time on green patches than on brown patches (56%, β _0_ = 0.19 ± 0.10, *z* = 1.90, *P* = 0.058), a preference that was not significantly difference from brown morphs (β = 0.09 ± 0.15, *z* = 0.62, *P* = 0.54 for the contrast to uniform brown morphs, Table 1). Green morphs, however, spend significantly more time on green than on brown patches (61%, β _0_ = 0.47 ± 0.14, *z* = 3.44, *P* = 0.00057), a preference that was significantly different from brown morphs (β = 0.38 ± 0.18, *z* = 2.09, *P* = 0.037, Table 1), but not-significantly different from lateral green morphs (β = 0.28 ± 0.17, *z* = 1.68, *P* = 0.093). Morphs thus ranked in increasing preference for green between abeyance in brown morphs over intermediate in lateral green morphs to a significant preference for green in green morphs.

**Table 1:** Generalized linear mixed model analysis of the preferences for green patches using binomial error distributions with logit link. Significant effects (at *P* ≤ 0.05) are shown in bold

All Data	b	SE	z	P
(Intercept)	0.097	0.121	0.80	0.42
Stage = Nymph	0.431	0.313	1.38	0.17
Sex = male	0.266	0.226	1.18	0.24
Morph = lateral green	0.094	0.153	0.62	0.54
Morph = green	**0.377**	**0.181**	**2.09**	**0.037**
Nymph * lateral green	0.448	0.475	0.94	0.35
Nymph * green	–0.912	1.488	–0.61	0.54
Male * lateral green	–0.029	0.296	–0.10	0.92
Male * green	–0.482	0.346	–1.40	0.16
Imagoes only	b	SE	*z*	*P*
(Intercept)	0.099	0.126	0.79	0.43
Sex = Male	0.331	0.246	1.35	0.18
Morph = lateral green	0.097	0.158	0.61	0.54
Morph = green	**0.377**	**0.186**	**2.03**	**0.043**
Male * lateral green	0.008	0.318	0.02	0.98
Male * green	–0.518	0.364	–1.42	0.15

There was some stage-specific variation, with nymphal stages tending to spend more time on green patches (β = 0.43 ± 0.31, *z* = 1.38, *P* = 0.17, Table 1). Males tended to spend more time on green backgrounds, but the difference to females was not significant (β = 0.27 ± 0.23, *z* = 1.18, *P* = 0.24, Table 1). Individual identity and date explained only a small amount of variation after accounting for fixed effects (LRT: R = 0.018 ± 0.012, χ ^2^_1_ = 2.59, *P* = 0.054 for the effect of individuals and *R* = 0.004 ± 0.006, χ ^2^_1_ = 0.33, *P* = 0.28 for the effect of date).

### Visual modeling analyses

**Figure 4:**
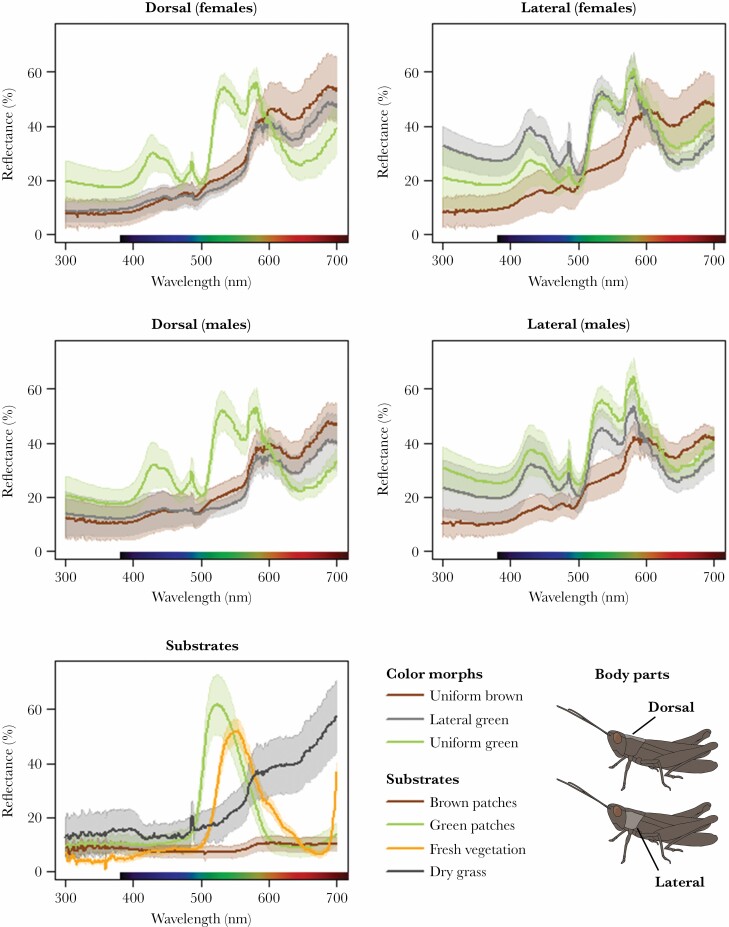
Average reflectance curves for the three color morphs (separated by sex and area of the pronotum), average reflectance curves for the brown and green substrates used in the choice experiment and samples from natural grassland vegetation. Average curves are based on samples from ten individuals and confidence intervals are shown as shaded areas.

Reflectance profiles differed among color morphs but less between the sexes (Figure 4). As expected, spectral profiles for the lateral green morph were more similar in overall appearance to green morphs on the lateral side and to brown morphs on the dorsal side (Figure 4). Sex differences were largely insignificant as compared to individual variation within morphs and below the discrimination threshold (both chromatically and achromatically) for all the six visual systems that we have modeled (Supplementary Tables S1 and S2). Similarly, different brown (or green) body parts were indistinguishable to all visual systems, both when comparing the dorsal and lateral sides of uniform individuals and when comparing the brown dorsal side of lateral green individuals to brown morphs or their lateral green side to green morphs (Table 2).

**Table 2:** **Chromatic distances (ΔS) and achromatic distances (ΔL) between colors of different morphs and body parts and as modelled using the visual models for six animal species as representatives of potential predators. Sexes were pooled in the analysis (see** **Supplementary Table S5** **for analyses separated by sex). Lateral green morphs are dorsally brown and laterally green and the column *Patch color* thus shows the color of the specific body parts. Delta values greater than 3 are shown in bold**

	Trichromatic species					
	Lizard		Spider		Bee	
	ΔS	ΔL	ΔS	ΔL	ΔS	ΔL
Brown vs. green body parts						
Brown vs. green morphs (dorsal view)	2.57	**5.11**	**6.50**	2.71	1.27	**6.18**
Brown vs. green morphs (lateral view)	2.11	**5.02**	**5.43**	**3.67**	2.76	**6.38**
Green vs. lateral green morphs (dorsal view)	1.74	**5.63**	**5.44**	**3.51**	0.43	**6.23**
Brown vs. Lateral green morphs (lateral view)	**3.21**	**5.11**	**6.75**	**3.46**	**3.82**	**6.79**
Lateral green morphs (dorsal vs. lateral side)	2.53	**6.74**	**5.90**	**4.79**	1.69	**7.88**
Brown vs. brown body parts						
Brown vs. lateral green morphs (dorsal view)	1.13	0.52	1.80	0.79	1.49	0.05
Brown morphs (dorsal vs. lateral side)	0.45	1.11	1.02	0.53	0.83	1.05
Green vs. green body parts						
Green vs. lateral green morphs (lateral view)	1.10	0.09	1.39	0.21	1.14	0.40
Green morphs (dorsal vs. lateral side)	0.32	1.01	1.05	1.49	1.03	1.25
	Tetrachromatic species					
	House fly		Starling		Peafowl	
	ΔS	ΔL	ΔS	ΔL	ΔS	ΔL
Brown vs. green body parts						
Brown vs. green morphs (dorsal view)	0.40	**6.25**	**5.91**	**3.50**	**5.79**	**3.16**
Brown vs. green morphs (lateral view)	1.97	**6.12**	**4.43**	**3.56**	**3.77**	**3.35**
Green vs. lateral green morphs (dorsal view)	0.91	**6.45**	**5.32**	**4.71**	**5.48**	**4.36**
Brown vs. Lateral green morphs (lateral view)	2.81	**6.52**	**5.67**	2.74	**4.75**	2.50
Lateral green morphs (dorsal vs. lateral side)	0.91	**7.94**	**5.53**	**4.90**	**5.31**	**4.58**
Brown vs. brown body parts						
Brown vs. lateral green morphs (dorsal view)	1.26	0.21	1.28	1.21	0.97	1.21
Brown morphs (dorsal vs. lateral side)	0.72	1.21	1.01	0.94	1.09	0.87
Green vs. green body parts						
Green vs. lateral green morphs (lateral view)	0.95	0.41	1.43	0.82	1.21	0.85
Green morphs (dorsal vs. lateral side)	0.87	1.08	1.08	1.01	1.06	1.07

**Figure 5:**
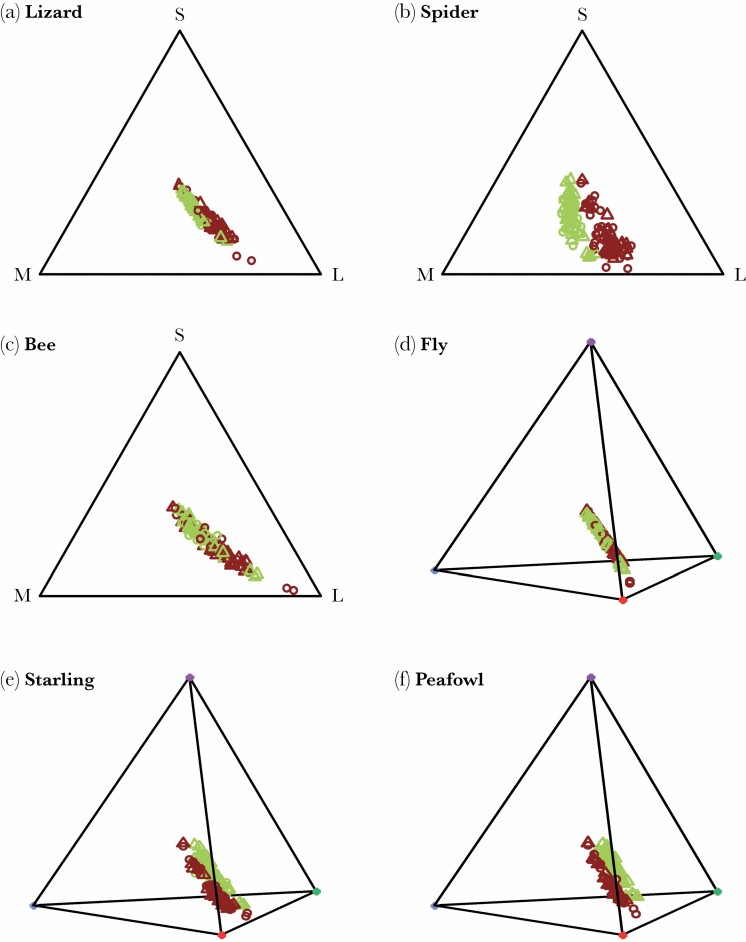
Color space plots of reflectance spectra of animal representative of potential predators. Each dot refers to one body area of one individual (60 individuals in total x 2 body areas) with females shown as triangles and males as circles. Colors show the body color of the respective body area (green = dorsal or lateral body parts of uniform green individual and lateral body parts of lateral green individuals, brown = dorsal or lateral body parts of uniform brown individual and dorsal body parts of lateral green individuals).

Green and brown body parts (between morphs or between sides in bicolored lateral green individuals) were predicted to be visually distinguishable to all representatives of potential predators, but there was significant variation among visual systems in how differences would be perceived (Table 2). The trichromatic jumping spider and the two tetrachromatic birds (European starling and peafowl) are predicted to perceive chromatic as well as achromatic differences between green and brown body parts (Figure 5, Table 2). In contrast, the trichromatic lizard and honey bee as well as the tetrachromatic house fly are predicted to perceive color differences largely achromatically (Figure 5, Table 2).

### Crypsis

Reflectance spectra of our experimental substrates were broadly similar to natural substrates with green patches resembling fresh vegetation (albeit blue-shifted) and brown patches resembling dry vegetation (albeit with lower overall reflectance) (Figure 4). However, all visual systems are predicted to perceive differences between fresh and dry vegetation, between green and brown experimental patches, and between natural and artificial substrates (ΔS and ΔL >3; Supplementary Figure S1, Supplementary Tables S1 and S2).

We compared the discriminability of grasshoppers against artificial and natural substrate backgrounds as seen by the six visual model species. Brown body parts are predicted to be discriminable against our artificial backgrounds, but not against dry grass from natural habitats (Supplementary Tables S3 and S4). Surprisingly, green body parts are predicted to be discriminable against both our artificial backgrounds and against fresh vegetation from natural habitats (Supplementary Tables S3 and S4). Brown body parts contrast strongly against green backgrounds (both artificial and fresh vegetations), in particular to the visual systems of the spider and the two birds in our set (Supplementary Tables S3 and S4). Green body parts contrast against brown backgrounds (both artificial and fresh vegetations), but more in luminance as compared to chromatically to most visual systems (Supplementary Tables S3 and S4).

## DISCUSSION

We tested for differential habitat choice in meadow grasshoppers of different color morphs. As expected for a grassland species we found an overall preference for green patches in the laboratory choice setting. This preference indicates that meadow grasshoppers may choose their microhabitat mainly by food-related cues when predators are absent. Nymphae and males tended to spend more time on green patches, although both contrasts were not significantly different from zero. The most striking pattern was, however, an association between body color and the strength of preference for green patches: brown morphs displayed the weakest preference that was not significantly different from random choice, bicolored (lateral green) morphs preferred uniformly green more clearly and green individuals showed the strongest preference for green. While differences are overall small and probably not sufficiently strong on their own, they might still contribute to some habitat segregation in natural habitats. When the color and brightness of the morphs was compared as perceived by vertebrate and invertebrate predators, two insects and a lizard would be able to discriminate morphs on the basis of brightness, and a spider and birds also on the basis of chroma. Although we find that brown morphs do not stand out from dry vegetation, green morphs do stand out to both dry and fresh vegetation. While our data do not demonstrate improved crypsis of green morphs, grasslands are very heterogenous and improved crypsis on average seems still possible.

The role of background matching choice behavior in homochromy has been studied a number of times in grasshoppers (Edelaar et al. 2019; Camacho et al. 2020), but its role in the maintenance of the green-brown polymorphism has not been investigated. By quantifying the phenotype of wild-caught individuals and linking this to their background settlement decisions our results add to other examples of phenotype-environment associations for color in arachnids (Bonte and Maelfait 2004), Lepidoptera (Eacock et al. 2019), crustaceans (Todd et al. 2006; Stevens et al. 2015; Green et al. 2019), amphibians (Lowe and Addis 2019), and birds (Stevens et al. 2017), and provides evidence for a mechanism that may help to maintain the balanced green-brown polymorphism in grasshoppers.

We assume that microhabitat choice serves the purpose of predator avoidance, although other factors, such as temperature preferences, might also contribute to habitat choice decisions (Pitt 1999; Stuart-Fox and Moussalli 2009). In the meadow grasshopper, it has been found that green individuals survive better on average possibly due to better crypsis in dense grasslands (Köhler and Renker 2006). However, altitudinal gradients in color morph ratios (Köhler et al. 2017) suggest a role of thermoregulation and indeed brown morphs of the species tend to have higher body temperatures than green individuals in natural populations (Köhler and Schielzeth 2020). In our laboratory situation, the two background colors did not differ in temperature, so that substrate choice does not convey a thermoregulatory advantage under laboratory conditions. Yet under natural conditions, the species may have evolved morph-specific solutions to the trade-off between crypsis and thermoregulation. Such trade-offs may explain why brown individuals behave indiscriminately rather than preferring brown backgrounds.

We analyzed the three most abundant color morphs of the meadow grasshopper, two of which are rather uniformly colored, while the lateral green morph shows a markedly bicolored pattern. This bicolored pattern, though not truly disruptive, exposes an individual’s shape less clearly and could thus impede detection by visual predators (Cuthill et al. 2005; Stevens and Merilaita 2009). In principle, this might lead to different habitat preferences or less marked preferences overall. However, our data shows that bicolored morphs were intermediate in their substrate preferences between the two uniformly colored morphs. Intermediate preferences might reflect that crypsis of lateral green individuals on different backgrounds depends on viewing angle (better matched to brown when viewed from above, but better matched to green when viewed from the side). Both viewing angles are ecologically relevant, since some of the predators, such as lizards and frogs, would mostly view from the side, while others, such as birds, would see mostly top-views. Additional experiments would be needed to test if bicolored individuals prefer small-scale heterogenous backgrounds rather than the uniform patches that we used here.

There are multiple possibilities how individuals achieve a match between their body color and the habitat background even in the absence of phenotypic plasticity (Akcali and Porter 2017). This might be realized by genetic linkage or pleiotropy of body color with color preference loci (direct genetic habitat choice, Akcali and Porter 2017), by self-referent color matching (Gillis 1982; Wennersten et al. 2012; Eacock et al. 2019), habitat imprinting (Van Belleghem et al. 2016; Akcali and Porter 2017), or by match-dependent displacement (Edelaar et al. 2008). Direct genetic habitat choice is possible in a species with genetic morph determination, although there is currently no direct evidence (possibly due to lack of dedicated studies) for genetically determined color preferences in grasshoppers. Individuals might use perception of their own color phenotype as a reference to achieve color matching (Gillis 1982; Wennersten et al. 2012; Eacock et al. 2019; Edelaar et al. 2019; Camacho et al. 2020). Such self-referent color matching seems possible in principle, since the eyes of grasshoppers are placed laterally so that individuals are able to see parts of their body as well as the background. Individuals might asses match also indirectly by using the rate of disturbance as a reliable indicated or match and might even imprint on microhabitats in which they are less disturbed. However, matching by displacement can be achieved even if individuals settle randomly, but are more often disturbed in unmatched locations, for example by predators to which they will be more conspicuous if they are unmatched to the background. Displacement can continue until individuals find themselves in a matched location with less disturbance where they will spend more time in total. Indeed, in some other grasshoppers, the choice of matching habitats depends on the presence of predators (Ahnesjö and Forsman 2006). However, in our laboratory situation, this mechanism is less likely, since there was no source of match-dependent disturbance.

Matching habitat choice can contribute to the maintenance of balanced polymorphisms because it will tend to equalize fitness differences between morphs in heterogeneous environments (Edelaar et al. 2008; Ravigné et al. 2009). Mechanisms that equalize fitness differences alone do not protect populations against the loss of color morphs by genetic drift or episodes of strong directional selection. However, background-matching habitat preferences could lead to negative frequency-dependent selection if there is density-dependent competition in different habitat places. Competition for food is unlikely to be the driving forces in the case of meadow grasshoppers. Competition for safe hiding places, if all individuals aggregate in matched microniches, might attract predators and/or facilitate the transmission of diseases and/or ectoparasites. Individuals that are able to use alternative microniches could benefit if they are rare, leading to negative-frequency dependent selection. This possibility has never been explored in grasshoppers.

Visual modeling showed that the difference between green and brown body coloration is chromatically visible to some species, but not to others. Various studies show that visual predators prefer conspicuous prey (Bond and Kamil 2002; Baños-Villalba et al. 2018). Our results show that potential predators like birds and visually hunting spiders are likely to perceive chromatic differences between green and brown morphs, while lizards and (at least some) insects are apparently unable to perceive chromatic differences among morphs (Table 2). However, even these species perceive the difference in overall brightness, thus in the achromatic component of body coloration. One of the species that we included in our visual modeling, the honey bee, has three chromatic receptor types that have similar sensitivities as the three receptors of grasshoppers (Briscoe and Chittka 2001). It is thus possible that grasshoppers perceive morph differences achromatically, even if not chromatically. Perception of achromatic differences can, in principle, be sufficient to elicit differential substrate choice.

The meadow grasshopper is a short-winged species (with long-winged individuals occurring in low frequencies) with limited dispersal abilities (Ingrisch and Köhler 1998). Therefore, color morph specific microhabitat preferences might be considered as individual ecological microniches that allow individuals of different color morphs to coexist locally so that the population as a whole can reach higher densities, balance fitness differences between color morphs, and explain the maintenance of a balanced polymorphism if there is frequency-dependent competition for microniches as we argue above. Since laboratory conditions excluded match-dependent disturbance and microhabitat imprinting might only have happened before capture in the field, the results suggest self-referent background matching or direct genetic determined preferences. Visual modeling suggests that self-referencing might work via achromatic rather than chromatic information. Overall, our results contribute to explaining two important phenomena, maintenance of balanced polymorphisms and homochromy, with respect to the widespread green-brown polymorphisms in Orthoptera, though effects appear to be too small to suffice as the only explanation.

## Supplementary Material

arab133_suppl_Supplementary_Materials

## Notes

Data availability: Analyses reported in this article can be reproduced using the data and scripts provided by Heinze et al. (2021).
