# Supplementary material for: Evidence for morph-specific substrate choice in a green-brown polymorphic grasshopper
Source: Behav Ecol. 2021 Dec 16;33(1):17–26. doi: 10.1093/beheco/arab133 (PMC8857936; doi:10.1093/beheco/arab133)

Evidence for morph-specific substrate choice in a green-brown polymorphic grasshopper

Pauline Heinze, Petra Dieker, Hannah M. Rowland & Holger Schielzeth

Supplementary Tables

Table S1: Comparisons of chromatic distances (ΔS) and achromatic distances (ΔL) between sexes, body parts and substrates as modelled using the visual models for a lizard, a jumping spider and the honey bee. 2

Table S2: Comparisons of chromatic distances (ΔS) and achromatic distances (ΔL) between sexes, body parts and substrates as modelled using the visual models for the house fly, European starling and peafowl. 3

Table S3: Chromatic distances (ΔS) and achromatic distances (ΔL) between color of grasshoppers and substrates as modelled using the visual models for a lizard, a jumping spider and the honey bee. 4

Table S4: Chromatic distances (ΔS) and achromatic distances (ΔL) between color of grasshoppers and substrates as modelled using the visual models for the house fly, European starling and peafowl. 5

Table S5: Chromatic distances (ΔS) and achromatic distances (ΔL) between colors of different color morphs as modelled using the visual models for six animal species as representatives of potential predators. 6

Supplementary Figures

Figure S1: Color space plots of reflectance spectra of natural habitats and experimental patches as seen by six different species. 7

Table S1: Comparisons of chromatic distances (ΔS) and achromatic distances (ΔL) between sexes, body parts and substrates as modelled using the visual models for a lizard, a jumping spider and the honey bee. Delta values greater than 3 are shown in bold.

	Lizard		Spider		Bee	
Comparison	ΔS	ΔL	ΔS	ΔL	ΔS	ΔL
**Visual distance between the sexes**						
Brown female vs. male (dorsal view)	1.73	0.11	**3.14**	0.49	2.62	1.05
Green female vs. male (dorsal view)	0.71	0.08	0.64	0.31	0.51	0.11
Lateral green female vs. male (dorsal view)	1.92	0.21	**3.11**	0.28	2.88	1.19
Brown female vs. male (lateral view)	0.54	0.09	1.55	0.33	0.98	0.51
Green female vs. male (lateral view)	1.57	2.05	1.97	1.53	1.61	2.48
Lateral green female vs. male (lateral view)	0.63	1.75	1.57	1.77	1.29	2.24
**Visual distance between the body parts**						
Dorsal vs. Lateral (brown females)	0.93	1.11	1.35	0.45	0.61	1.33
Dorsal vs. Lateral (green females)	0.65	0.02	1.16	0.54	0.56	0.01
Dorsal vs. Lateral (lateral green females)	**3.55**	**7.53**	**7.54**	**5.38**	**3.46**	**9.29**
Dorsal vs. Lateral (brown males)	0.56	1.10	1.34	0.62	1.50	0.79
Dorsal vs. Lateral (green males)	0.43	1.94	1.35	2.38	1.51	2.36
Dorsal vs. Lateral (lateral green males)	1.21	**5.56**	**4.06**	**3.89**	0.78	**5.85**
**Visual distances between substrates**						
Artificial green vs. artificial brown	**6.44**	**9.80**	**10.81**	**7.47**	**9.37**	**7.81**
Artificial green vs. fresh vegetation	**3.89**	**3.18**	**6.63**	2.27	**4.56**	**3.74**
Artificial green vs. dry grass	**4.65**	1.41	**11.73**	2.32	**5.67**	0.82
Artificial brown vs. fresh vegetation	**8.75**	**6.62**	**12.09**	**5.20**	**13.7**	**4.08**
Artificial brown vs. dry grass	**3.81**	**8.39**	**4.94**	**9.79**	**3.70**	**6.99**
Fresh vegetation vs. dry grass	**5.39**	1.77	**10.09**	**4.59**	**10.05**	2.91

Table S2: Comparisons of chromatic distances (ΔS) and achromatic distances (ΔL) between sexes, body parts and substrates as modelled using the visual models for the house fly, European starling and peafowl. Delta values greater than 3 are shown in bold.

	House fly		Starling		Peafowl	
Comparison	ΔS	ΔL	ΔS	ΔL	ΔS	ΔL
**Visual distance between the sexes**						
Brown female vs. male (dorsal view)	2.06	0.90	2.37	1.02	1.62	1.07
Green female vs. male (dorsal view)	0.48	0.24	1.00	0.46	1.02	0.49
Lateral green female vs. male (dorsal view)	2.34	1.14	2.53	1.28	1.77	1.33
Brown female vs. male (lateral view)	0.71	0.47	1.23	0.28	0.99	0.33
Green female vs. male (lateral view)	1.38	2.55	2.10	0.79	1.85	0.74
Lateral green female vs. male (lateral view)	1.00	2.10	1.17	1.16	0.70	1.13
**Visual distance between the body parts**						
Dorsal vs. Lateral (brown females)	0.31	1.43	1.27	0.58	1.29	0.51
Dorsal vs. Lateral (green females)	0.39	0.16	1.39	0.38	1.43	0.45
Dorsal vs. Lateral (lateral green females)	2.33	**9.26**	**6.73**	**4.85**	**6.10**	**4.50**
Dorsal vs. Lateral (brown males)	1.33	1.00	1.11	1.33	1.05	1.26
Dorsal vs. Lateral (green males)	1.27	2.15	1.12	1.63	0.88	1.68
Dorsal vs. Lateral (lateral green males)	1.03	**6.02**	**4.17**	**4.97**	**4.31**	**4.70**
**Visual distances between substrates**						
Artificial green vs. artificial brown	**8.37**	**9.32**	**9.55**	**13.57**	**10.25**	**13.06**
Artificial green vs. fresh vegetation	**3.43**	**5.12**	**7.84**	0.70	**7.62**	0.41
Artificial green vs. dry grass	**5.55**	2.44	**11.2**	1.82	**12.53**	1.14
Artificial brown vs. fresh vegetation	**11.78**	**4.20**	**10.7**	**12.87**	**8.33**	**12.66**
Artificial brown vs. dry grass	2.84	**6.88**	**5.04**	**11.75**	**4.43**	**11.92**
Fresh vegetation vs. dry grass	**8.97**	2.67	**8.43**	1.12	**7.61**	0.73

Table S3: Chromatic distances (ΔS) and achromatic distances (ΔL) between color of grasshoppers and substrates as modelled using the visual models for a lizard, a jumping spider and the honey bee. Delta values greater than 3 are shown in bold. D = dorsal side, L = lateral side.

Body part	Substrate	Lizard		Spider		Bee	
		ΔS	ΔL	ΔS	ΔL	ΔS	ΔL
Brown body parts vs. "brown" substrates							
Brown morph (D)	Artificial brown	**4.79**	**8.02**	**7.94**	**9.01**	**6.39**	**5.59**
Brown morph (D)	Dry grass	1.05	0.37	**3.24**	0.79	2.91	1.40
Brown morph (L)	Artificial brown	**4.49**	**9.13**	**7.80**	**9.54**	**7.08**	**6.63**
Brown morph (L)	Dry grass	0.97	0.74	**3.49**	0.26	**3.70**	0.36
Lateral green (D)	Artificial brown	**3.75**	**7.50**	**6.16**	**8.21**	**4.94**	**5.54**
Lateral green (D)	Dry grass	0.08	0.88	1.49	1.58	1.71	1.45
Green body parts vs. "green" substrates							
Green morph (D)	Artificial green	**4.44**	**3.34**	**6.42**	**4.25**	**4.65**	**3.95**
Green morph (D)	Fresh vegetation	**6.39**	**6.52**	**7.21**	**6.52**	**8.57**	**7.69**
Green morph (L)	Artificial green	**4.64**	**4.35**	**7.44**	**5.74**	**5.23**	**5.21**
Green morph (L)	Fresh vegetation	**6.41**	**7.53**	**8.00**	**8.01**	**9.39**	**8.94**
Lateral green (L)	Artificial green	**5.36**	**4.44**	**7.46**	**5.52**	**6.37**	**5.61**
Lateral green (L)	Fresh vegetation	**7.46**	**7.62**	**9.05**	**7.79**	**10.52**	**9.35**
Brown body parts vs. "green" substrates							
Brown morph (D)	Artificial green	**4.16**	1.78	**11.85**	1.53	**3.39**	2.22
Brown morph (D)	Fresh vegetation	**4.36**	1.40	**8.39**	**3.80**	**7.34**	1.51
Brown morph (L)	Artificial green	**3.85**	0.67	**10.88**	2.06	**3.22**	1.18
Brown morph (L)	Fresh vegetation	**4.45**	2.51	**7.42**	**4.33**	**6.78**	2.56
Lateral green (D)	Artificial green	**4.70**	2.29	**11.38**	0.74	**4.78**	2.28
Lateral green (D)	Fresh vegetation	**5.46**	0.89	**8.98**	**3.01**	**8.83**	1.46
Green body parts vs. "brown" substrates							
Green morph (D)	Artificial brown	2.36	**13.13**	**5.05**	**11.72**	**5.27**	**11.76**
Green morph (D)	Dry grass	1.78	**4.75**	**5.43**	1.93	2.13	**4.77**
Green morph (L)	Artificial brown	2.39	**14.15**	**4.12**	**13.21**	**4.34**	**13.02**
Green morph (L)	Dry grass	1.56	**5.76**	**4.57**	**3.42**	1.14	**6.03**
Lateral green (L)	Artificial brown	1.30	**14.24**	**3.42**	**13.00**	**3.27**	**13.42**
Lateral green (L)	Dry grass	2.59	**5.85**	**5.41**	3.2	1.26	**6.43**

Table S4: Chromatic distances (ΔS) and achromatic distances (ΔL) between color of grasshoppers and substrates as modelled using the visual models for the house fly, European starling and peafowl. Delta values greater than 3 are shown in bold. D = dorsal side, L = lateral side.

		House fly		Starling		Peafowl	
Body part	Substrate	ΔS	ΔL	ΔS	ΔL	ΔS	ΔL
Brown body parts vs. "brown" substrates							
Brown morph (D)	Artificial brown	**4.92**	**5.74**	**6.74**	**12.23**	**5.08**	**12.38**
Brown morph (D)	Dry grass	2.23	1.13	2.34	0.48	0.91	0.46
Brown morph (L)	Artificial brown	**5.59**	**6.95**	**6.44**	**13.17**	**4.35**	**13.26**
Brown morph (L)	Dry grass	2.94	0.07	2.67	1.42	1.26	1.33
Lateral green (D)	Artificial brown	**3.68**	**5.54**	**5.51**	**11.02**	**4.30**	**11.18**
Lateral green (D)	Dry grass	1.16	1.34	1.46	0.73	0.25	0.75
Green body parts vs. "green" substrates							
Green morph (D)	Artificial green	**3.87**	2.67	**5.99**	2.15	**7.39**	2.48
Green morph (D)	Fresh vegetation	**7.26**	**7.79**	**6.52**	2.86	**5.02**	2.88
Green morph (L)	Artificial green	**4.62**	**3.75**	**6.91**	**3.16**	**8.41**	**3.55**
Green morph (L)	Fresh vegetation	**8.04**	**8.86**	**6.81**	**3.86**	**5.21**	**3.95**
Lateral green (L)	Artificial green	**5.57**	**4.15**	**6.52**	2.34	**7.91**	2.69
Lateral green (L)	Fresh vegetation	**8.98**	**9.27**	**7.92**	**3.04**	**5.96**	**3.10**
Brown body parts vs. "green" substrates							
Brown morph (D)	Artificial green	**3.53**	**3.58**	**11.52**	1.34	**12.55**	0.68
Brown morph (D)	Fresh vegetation	**6.91**	1.54	**7.44**	0.64	**7.31**	0.27
Brown morph (L)	Artificial green	**3.01**	2.37	**10.62**	0.40	**11.48**	0.19
Brown morph (L)	Fresh vegetation	**6.32**	2.75	**6.74**	0.30	**6.53**	0.60
Lateral green (D)	Artificial green	**4.77**	**3.78**	**11.17**	2.56	**12.47**	1.89
Lateral green (D)	Fresh vegetation	**8.17**	1.33	**8.03**	1.86	**7.68**	1.48
Green body parts vs. "brown" substrates							
Green morph (D)	Artificial brown	**4.55**	**11.99**	**4.61**	**15.73**	**3.74**	**15.54**
Green morph (D)	Dry grass	1.84	**5.11**	**5.45**	**3.98**	**5.51**	**3.62**
Green morph (L)	Artificial brown	**3.75**	**13.07**	**3.94**	**16.73**	**3.24**	**16.61**
Green morph (L)	Dry grass	0.98	**6.19**	**4.45**	**4.99**	**4.47**	**4.69**
Lateral green (L)	Artificial brown	2.81	**13.47**	**3.32**	**15.92**	2.92	**15.76**
Lateral green (L)	Dry grass	0.46	**6.60**	**5.44**	**4.17**	**5.36**	**3.83**

Table S5: Chromatic distances (ΔS) and achromatic distances (ΔL) between colors of different color morphs as modelled using the visual models for six animal species as representatives of potential predators. Lateral green morphs are dorsally brown and laterally green and the column *Patch color* thus shows the color of the specific body parts. Delta values greater than 3 are shown in bold.

Trichromatic species	Patch color	Lizard ΔS	Lizard ΔL	Spider ΔS	Spider ΔL	Bee ΔS	Bee ΔL
Females dorsal							
Brown vs. Green	Brown vs. green	**3.05**	**5.13**	**7.53**	2.62	2.39	**6.66**
Brown vs. Lateral green	Brown vs. brown	1.20	0.55	2.04	0.92	1.57	0.03
Green-vs. Lateral green	Green vs. brown	2.11	**5.68**	**6.00**	**3.54**	0.83	**6.69**
Males dorsal							
Brown vs. Green	Brown vs. green	2.14	**5.10**	**5.64**	2.81	0.81	**5.72**
Brown vs. Lateral green	Brown vs. brown	1.35	0.45	2.04	0.71	1.83	0.11
Green-vs. Lateral green	Green vs. brown	1.26	**5.55**	**4.91**	**3.52**	1.79	**5.61**
Females lateral							
Brown vs. Green	Brown vs. green	1.51	**3.99**	**5.17**	2.72	2.47	**5.33**
Brown vs. Lateral green	Brown vs. green	**3.70**	**5.86**	**8.07**	**4.01**	**4.76**	**7.93**
Green-vs. Lateral green	Green vs. green	2.20	1.87	**3.05**	1.29	2.47	2.60
Males lateral							
Brown vs. Green	Brown vs. green	2.55	**5.95**	**5.53**	**4.58**	2.99	**7.30**
Brown vs. Lateral green	Brown vs. green	2.56	**4.02**	**5.07**	2.56	2.61	**5.18**
Green-vs. Lateral green	Green vs. green	0.02	1.93	0.49	2.01	0.52	2.12

Tetrachromatic species	Patch color	House fly ΔS	House fly ΔL	Starling ΔS	Starling ΔL	Peafowl ΔS	Peafowl ΔL
Females dorsal							
Brown vs. Green	Brown vs. green	1.37	**6.59**	**6.50**	**3.23**	**6.10**	2.87
Brown vs. Lateral green	Brown vs. brown	1.28	0.24	1.37	1.19	1.02	1.19
Green-vs. Lateral green	Green vs. brown	0.50	**6.83**	**5.60**	**4.42**	**5.59**	**4.07**
Males dorsal							
Brown vs. Green	Brown vs. green	0.44	**5.92**	**5.41**	**3.79**	**5.48**	**3.46**
Brown vs. Lateral green	Brown vs. brown	1.58	0.00	1.62	1.45	1.23	1.44
Green-vs. Lateral green	Green vs. brown	1.97	**5.93**	**4.98**	**5.24**	**5.23**	**4.90**
Females lateral							
Brown vs. Green	Brown vs. green	1.77	**5.00**	**4.14**	**3.02**	**3.57**	2.81
Brown vs. Lateral green	Brown vs. green	**3.53**	**7.59**	**6.67**	**3.08**	**5.48**	2.80
Green-vs. Lateral green	Green vs. green	2.05	2.59	2.94	0.06	2.44	0.02
Males lateral							
Brown vs. Green	Brown vs. green	2.17	**7.08**	**4.65**	**4.09**	**3.95**	**3.88**
Brown vs. Lateral green	Brown vs. green	1.85	**5.02**	**4.42**	2.19	**3.86**	1.99
Green-vs. Lateral green	Green vs. green	0.37	2.06	0.41	1.89	0.15	1.89

Figure S1: Color space plots of reflectance spectra of natural habitats and experimental patches as seen by six different species.

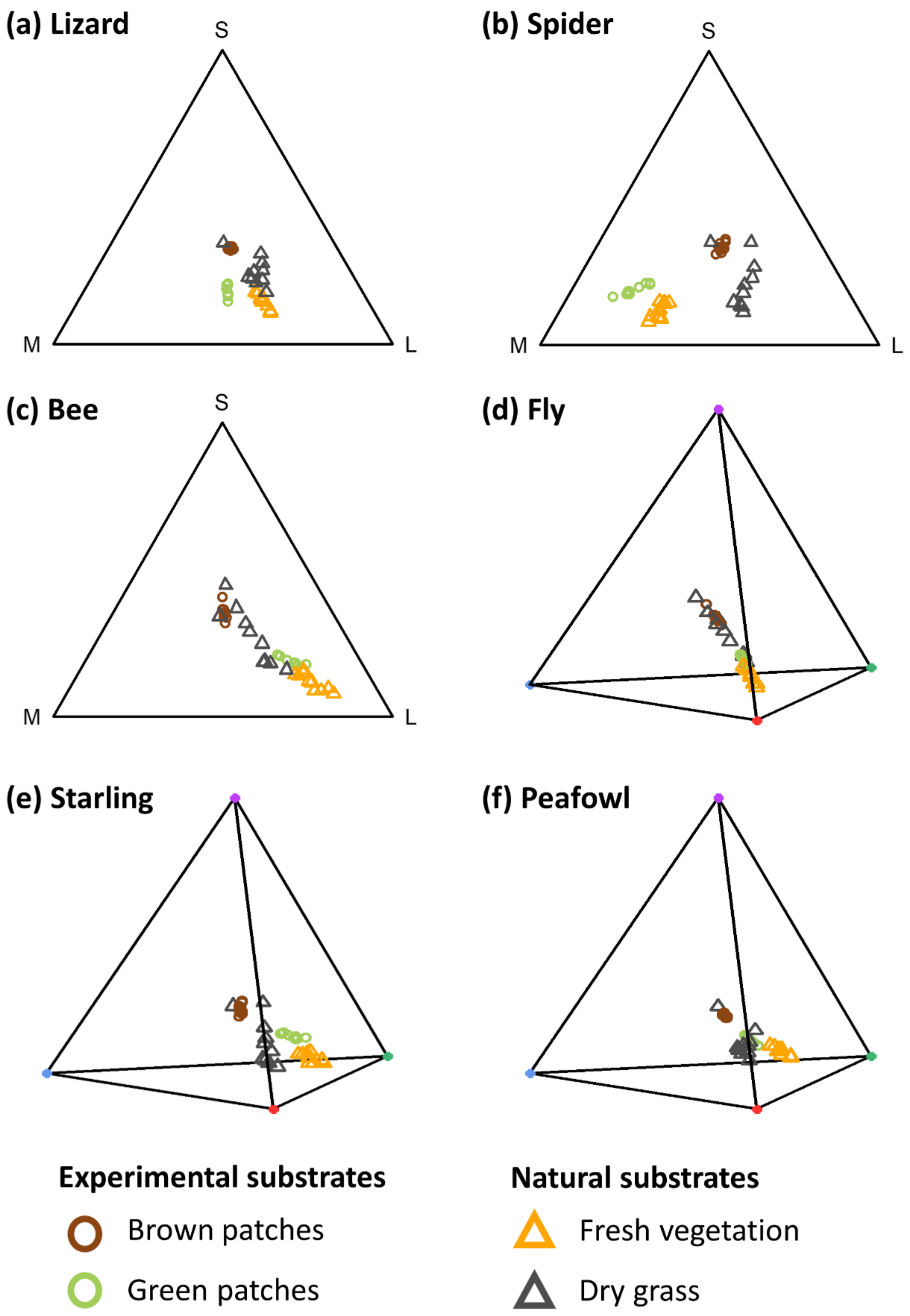

Supplement: arab133_suppl_Supplementary_Materials [file arab133_suppl_supplementary_materials.pdf]
